# Chemical Composition of 21 Cultivars of Sour Cherry (*Prunus cerasus*) Fruit Cultivated in Poland

**DOI:** 10.3390/molecules25194587

**Published:** 2020-10-08

**Authors:** Anna Sokół-Łętowska, Alicja Z. Kucharska, Grzegorz Hodun, Marta Gołba

**Affiliations:** 1 Departament of Fruit, Vegetable and Plant Nutraceutical Technology, Wroclaw University of Environmental and Life Sciences, Chełmońskiego 37, 51-630 Wrocław, Poland; alicja.kucharska@upwr.edu.pl (A.Z.K.); marta.golba@upwr.edu.pl (M.G.); 2 Department of Pomology, Gene Resources and Nurseries, Research Institute of Horticulture in Skierniewice, ul. Pomologiczna 18, 96-100 Skierniewice, Poland; grzegorz.hodun@inhort.pl

**Keywords:** sour cherry, phenolic compounds, antioxidant capacity, organic acids, sugars

## Abstract

Sour cherry (*Prunus cerasus* L.) is a very important fruit crop for producers as well as consumers. To obtain information on sour cherry fruit, we determined the sugar and organic acid composition and phenolic compound contents of twenty-one cultivars and genotypes of *Prunus cerasus* L. by HPLC quantification. Antioxidant capacity was determined by DPPH radical scavenging, reducing power (FRAP) and determination of substances reacting with Folin–Ciocalteu reagent (FCRS). The main organic acids in sour cherries were malic and malonic acids, and the main sugars were glucose and fructose. The highest sugar content was found in the “Lucyna” cultivar and the highest organic acids in “Paraszt Meggy” and “Suda Hardy”. The richest in phenolic compounds were “Wieluń 17”, “Sokówka Nowotomyska”, “Grosenkirch” “Sokówka Nowotomyska” “Grosenkirch” (anthocyanins, flavanols and total phenolics), and “Meteor” (phenolic acids). Flavan 3-ols were not quantified in every cultivar. “Dradem, “Turgieniewka”, “Wróble”, and “Nana” contained the smallest amounts of phenolic compounds. Antioxidant capacity was highly correlated with phenolic compound composition. According to principal component analysis (PCA), it was concluded that cultivars whose harvest maturity was medium to late contained more flavonols and anthocyanins and were characterized by higher antioxidant capacity than those whose harvest maturity was classified as early or early to medium.

## 1. Introduction

The *Rosaceae* family includes many species, among which cherries are one of the most popular in homes and industrial processing.

Sour cherry (*Prunus cerasus* L.), belonging to the *Rosaceae* family, is one of the most popular fruits, which is widely used both fresh and processed. Tart cherries are used for the home or industrial production of juices, canned fruit, brandy, liqueurs, preserves, etc. The fruit is characterized by a sour taste, juicy flesh, color from light red to dark red, and a pleasant aroma. The potential health benefits of tart cherries are quite well documented. In traditional medicine, they have been used as prophylactic agents against cardiovascular damage, Alzheimer’s disease, inflammatory diseases, and chronic ailments marked by elevation in oxidative-stress such as cancer and diabetes. Cherries improve appetite, lower blood pressure, protect against oxidative stress, reduce pain and muscle damage caused by exercise, modulate blood glucose and reduce inflammation [1,2,3]. These beneficial advantages of tart cherries are due to the high content of antioxidant compounds, which play an important role as promoters of human health.

The chemical composition of sour cherry fruit is determined by the cultivar as well as climate and soil conditions [4]. According to the literature, the fruit of tart cherry contains 8.0–21.5 g/100 g fresh weight (FW) of sugars, mainly glucose, fructose and sucrose, and from 295.0 to 1742.0 mg/100 g FW of organic acids, mainly malic acid. Cherries contain 254.0–407.0 mg of total polyphenol/100 g FW [5]. The main phenolic acids are 3-caffeoylquinic, 5-caffeoylquinic and *p*-coumaric acids. Flavanols (catechin and epicatechin derivatives) and flavonols (glycosides of quercetin and kaempferol) are mainly identified in fruit. The red color of tart cherries is a result of presence of anthocyanins. The antioxidant capacity of sour cherries is between 200.0 and 2000.0 µmol TE/100 g FW [2,6,7,8,9,10].

Sour cherry (*Prunus cerasus* L.) is a very important fruit crop in Poland. The production of sour cherries in 2018 was over 200 thousand tonnes [11]. Differentiation of constituents among sour cherry cultivars is important for the evaluation of fruit quality for producers as well as for consumers. It can also be very significant for health-connected features of tart cherries. In the available literature, there are only a few studies comparing the composition and antioxidant capacity of more than five varieties and/or cultivars [6,7,8,10,12,13]. Contents of total phenolics were analyzed by the Folin–Ciocalteu method in the majority of these studies.

The aim of this study was the comparison of 21 sour cherry cultivars which broadens the knowledge about the diversity of composition of bioactive compounds in cherries and reveals cultivars with high levels of phenolics and other constituents. A number of compounds that are important for sensory and health-related quality are determined in the study, providing an integrated approach of the chemical composition of cherries. Polyphenols, organic acids, sugars content and antioxidant capacity in twenty-one cultivars and genotypes of sour cherry fruit were compared. The fruits originated from known cultivars and local genotypes of different fruit color and maturity time, cultivated in the Research Institute of Horticulture in Skierniewice, Poland.

Relationships among studied parameters and cultivars were investigated by the correlations and principal component analysis (PCA).

## 2. Results

Characteristics of cultivars considered in this study are given in the Material and Method section. Eighteen cultivars and three local genotypes were tested in the study. The evaluated genotypes differed in fruit size, skin and juice color and in the time of fruit ripening.

Weights of fruit were from 2.7–3.2 g (“Marasca”) to 5.5-5.9 g (“Grosenkirch”), skin color was from light red (“Dradem”, ‘Malinówka”, “Meteor”, Montmorency” and “Wróble”) to almost black (“Demesova”) and juice color from colorless in “Montmorency” to very dark red in “Demesova”. Harvest maturity was described as:

- early to medium—”Krasnaja Płodorodnaja” and “Montmorency”,
- medium—”Demesova”, Granatnaja”, “Paraszt Meggy”, “Turgieniewka” and “Zagoriewskaja”,
- medium to late—”Dradem”, “Lucyna”, “Marasca”, “Mołodiożnaja”, “Sokówka Nowotomyska” and “Wróble”,
- late—”Grosenkirch”, “Malinówka”, “Meteor”, “Nana”, “Sokówka nr 9”, “Suda Hardy” and “Wieluń 17”,
- very late—”Sokówka nr 6”.

In our study antioxidant capacity measured by determining substances reacting with Folin–Ciocalteu reagent, reducing power FRAP and DPPH radical scavenging activity was performed and the chromatographic profiles of sugars, organic acids and phenolic compounds such as anthocyanins, phenolic acids, flavonols and flavan 3-ols were established. 

### 2.1. Sugars

Sugar content is a very important attribute in sensory evaluation, and it is decisive for the acceptance of the sour cherry fruit taste. Generally, the higher the sugar content in the fruit, the less sour the taste. Free sugars were determined by high-performance liquid chromatography (HPLC) and presented in Table 1. Total sugars (TS) were from 8.62 g/100 g FW in the “Turgieniewka” cultivar to 15.38 g/100 g FW in the “Lucyna” cultivar (Table 1). It was similar to sugar content in other sour fruit, for example, cornelian cherry, in which total sugars in the fruits were 4.1–16.4% [14,15] and less than in sweet cherries, where 7.7–26.5% of total sugars was determined [16,17,18]. Sour cherries can contain from 6.0 g/100 g FW to 16.33 g/100 g FW of carbohydrates [19,20]. As in many other fruits, in carbohydrate composition, glucose and fructose were dominant and represented 54.8–78.7% of all sugars. Glucose was found from 2.81 g/100 g FW in “Wieluń 17” to 5.68 g/100 g FW in “Lucyna”, and it was the dominant sugar in most cultivars. Fructose was dominant only in “Meteor” (4.88 g/100 g FW), “Montmorency” (4.22 g/100 g FW) and “Wieluń 17” (3.68 g/100 g FW). Fructose content was determined from 2.74 g/100 g FW (“Granatnaja”) to 4.88 g/100 g FW (“Meteor”). In Hungarian cultivars, Papp et al. [12] determined 6.06-9.08 g/100 g FW of glucose and 3.54-4.91 g/100 g FW of fructose. Sorbitol content varied from 1.93 g/100 g FW (“Nana” and “Turgieniewka” to 3.12 g/100 g FW (“Meteor”). Saccharose was determined in concentration from 1.03 g/100 g FW (“Nana”) to 1.41 g/100 g FW (“Demesova”), but not in all cultivars. In “Grosenkirch”, “Marasca”, “Montmorency”, “Paraszt Meggy” and “Turgieniewka” cultivars saccharose was not detected. Xylose (1.30–1.36 g/100 g FW) was determined only in three cultivars, i.e., “Granatnaja”, “Sokówka Nowotomyska” and “Wróble”, as was galactose (1.39–1.53 g/100 g FW), detected only in “Lucyna”, “Meteor” and “Mołodiożnaja”.

### 2.2. Organic Acids

Organic acids are fundamental to the typical sour cherry taste, which distinguishes it from sweet cherries. Total acid (TA) content (Table 2) was between 2300.5 mg/100 g FW and 1294.4 mg/100 g FW. The cultivars “Paraszt Meggy”, “Suda Hardy”, “Wróble” and “Krasnaja Płodorodnaja” contained more than 2200 mg/100 g FW. “Malinówka”, “Marasca” and “Montmorency” contained less than 1600 mg/100 g FW of total organic acids. The organic acid content in sour cherry is similar to or less than in cornelian cherry (*Cornus mas* L.), in which 1.7–4.6% organic acid content was determined [14,15], and in Japanese quince, which contains 3.5–4.5% organic acids [21]. Sweet cherries contain 0.6–3.7% organic acids [18].

In the sour cherry genotypes studied, five organic acids were identified and quantified: malic, oxalic, malonic, shikimic and fumaric. The main organic acid in sour cherry was malic acid, whose content ranged from 1027.4 mg/100 g FW to 1976.2 mg/100 g FW and accounted for 78.2–88.3% of total organic acids. The second acid according to content was malonic acid (11.2–21.3% in TA) and its content ranged from 231.5 mg/100 g FW in the “Turgieniewka” cultivar to 406.5 mg/100 g FW in the “Mołodiożnaja” cultivar. Oxalic, shikimic and fumaric acids were determined in much lower concentrations; these acids together accounted for 0.4–1.4% of total sour cherry organic acids. The profile and content of organic acids are confirmed by other studies [12,13,17,18].

An important index for estimation of the organoleptic properties and consumer”s acceptability of sour cherry fruit is the Total Sugar/Titratable Acidity (TS/TA) ratio [2,6,22]. For the investigated sour cherry the TS/TA ratio varied from 4.17 for the “Turgieniewka” cultivar to 7.95 for the “Montmorency” cultivar. Research performed by Wojdyło et al. [6], Najafzadeh et al. [8] and Grafe and Schuster [13] showed that the TS/TA ratio for sour cherries was from 3.4 to 19.5.

For other fruits, which are sweeter than tart cherries, the sweetness/sourness TS/TA ratio is higher. For Idared apples the TS/TA ratio was 19.7–28.5 [23], for apricots 8.4–9.2 [24], but for pomegranates and cornelian cherry it was 2.3–7.0 [14,25]. This acceptability index in sweet cherries can be above the value of 25 [18].

### 2.3. Phenolic Compounds

Phenolic compound content in sour cherry fruit was investigated by many authors [2,6,7,18,26,27,28,29,30,31,32,33]. In our study anthocyanins, phenolic acids, flavonols and flavanols concentration were quantified (Table 3).

**Figure 1:**
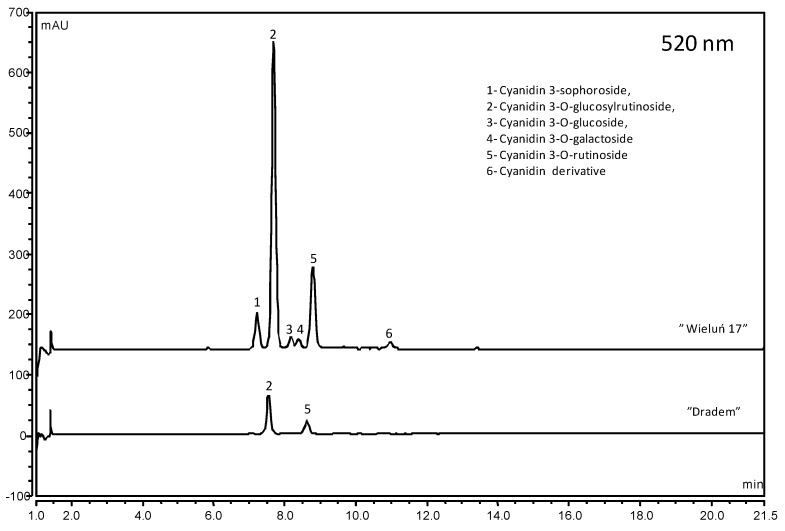
Chromatograms (520 nm) of sour cherry fruit cultivars with the highest and the lowest content of anthocyanins.

Fruit color was from light red to almost black and depended on the anthocyanin content, which was from 17.97 mg/100 g FW in “Dradem” to 131.28 mg/100 g FW in “Wieluń 17” (Figure 1). In addition to the above-mentioned cultivars, “Demesova”, “Sokówka Nowotomyska” and “Grosenkirch” contained more than 120 mg/100 g FW. “Montmorency”, “Wróble” and “Nana” were the cultivars with the content of anthocyanins lower than 50 mg/100 g FW. According to data reported by other authors anthocyanin content in sour cherries was from 21.0 to 285.0 mg/100 g FW [7], from 11.3 to 93.5 mg/100 g FW [12], from 65.1 to 82.4 mg/100 g FW [18], from 45.0 to 109.0 mg/100 g FW [22] and from 2.7 to 28.0 mg/100 g FW [29]. Sweet cherry fruit can contain from 1.2 to 900.0 mg/100 g FW [16,17,30]. Other red stone fruits, such as cornelian cherry, contain, depending on the cultivar, 29.2–341.2 mg anthocyanins in 100 g FW [15,31].

The main anthocyanins are: cyanidin 3 *O*-(2′glucosyl) rutinoside, and cyanidin 3 *O*-rutinoside; on average, these anthocyanins accounted for 62.8% and 27.0% of all anthocyanins (Supplementary Materials Table S1).

In most cultivars, cyanidin 3-*O*-(2′glucosyl) rutinoside was dominant, but it was absent in the “Demesova” and “Granatnaja” cultivars, in which cyanidin 3 *O*-rutinoside was the main anthocyanin and represented over 93% of total anthocyanins. Cyanidin 3 *O*-sophoroside, cyanidin 3 *O*-glucoside, and cyanidin 3 *O*-sambubioside 5-rutinoside were on average 5.2%, 1.8%, and 1.3%, respectively. In “Demesova”, there was no cyanidin 3 *O*-sambubioside 5-rutinoside but cyanidin 3 *O*-glucoside comprised 5.5% of all anthocyanins (in other cultivars cyanidin 3 *O*-glucoside accounted for 0.6–3.3% of total anthocyanins). Differentiation of the sour cherry anthocyanin profile was reported previously by Simunic et.al. [29], Blando et al. [32], Homoki et al. [33] and Filimon et al. [34].

**Figure 2:**
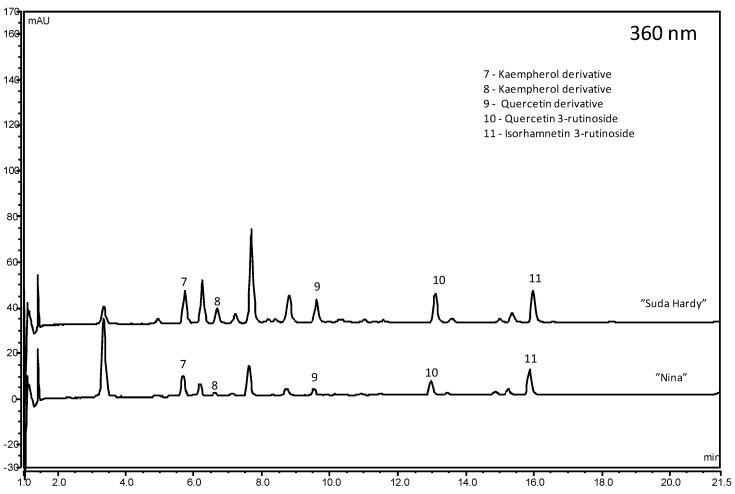
Chromatograms (360 nm) of sour cherry fruit cultivars with the highest and the lowest content of flavonols.

The second group of phenolic compounds present in sour cherries is flavonols. Flavonols, similarly to anthocyanins, strongly influence the antioxidant activity and play an important role in the health benefits of sour cherries. Total flavonol content was between 10.41 mg/100 g FW and 25.08 mg/100 g FW (Figure 2). In the examined cherries the highest concentration, above 20 mg/100 g FW, was determined in “Suda Hardy”, “Sokówka Nowotomyska”, “Marasca” and “Wieluń 17” cultivars, and the lowest, under 14 mg/100 g FW, in “Wróble”, “Zagoriewskaja”, “Nana”, “Krasnaja Płodorodnaja”, and “Mołodiożnaja” cultivars. According to many authors [18,35], quercetin, kaempferol and isorhamnetin rutinosides and glucosides are dominant in different proportions in sour cherry cultivars (Supplementary Material Table S2). According to other studies, in sour cherries, flavonols are at the level of 2.6–8.3 mg/100 g FW [2,27,35].

**Figure 3:**
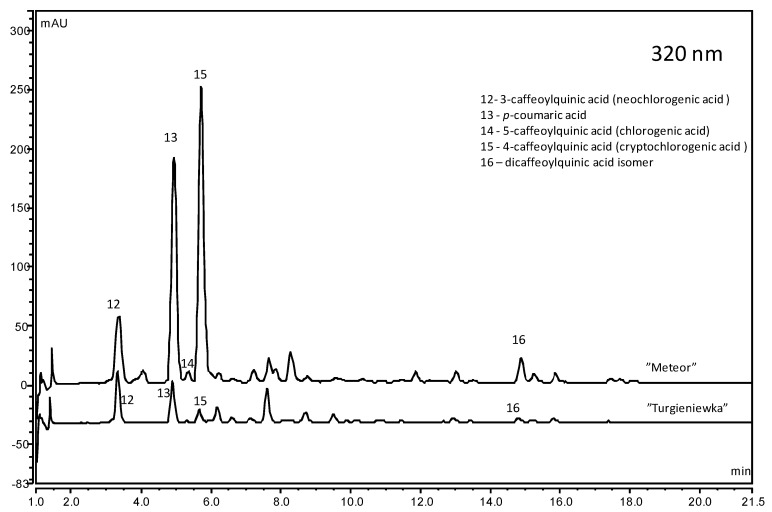
Chromatograms (320 nm) of sour cherry fruit cultivars with the highest and the lowest content of phenolic acids.

The next important group of phenolic compounds present in sour cherries is phenolic acids, which were determined at the level from 16.56 mg/100 g FW in “Turgieniewka” to 76.25 mg/100 g FW in “Grosenkirch”, and up to 126.99 mg/100 g FW in the “Meteor” cultivar (Figure 3).

Neochlorogenic, chlorogenic and *p*-coumaric acids were dominant in sour cherry fruit (Supplementary Material Table S3). A review on sour cherry fruits [2] reported 42.4 mg of phenolic acids per 100 g FW, but Wojdyło et al. [6] found a phenolic acid content from 39 to 263 mg/100 g FW (259–1200 mg/100 g DW). In the cultivars “Dradem”, “Lucyna” and “Turgieniewka” Wojdyło et al. [6] determined 218 mg/100 g FW, 118 mg/100 g FW and 168 mg/100 g FW of phenolic acids respectively, and in our research, these three cultivars contained 44.00 mg/100 g FW, 30.94 mg/100 g FW, and 16.56 mg/100 g FW respectively. In sweet cherries, 3.4–123.2 mg/100 g FW of phenolic acids was determined [2,16,27] and in cornelian cherry, from 10.9 to 37.5 [15].

**Figure 4:**
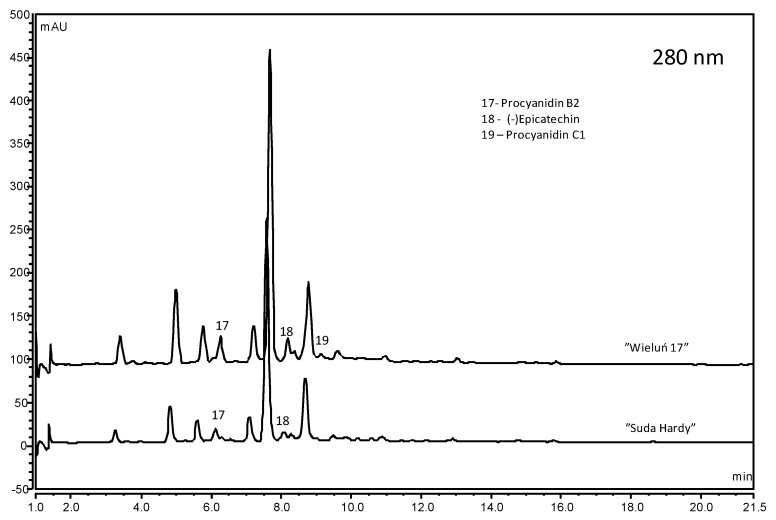
Chromatograms (280 nm) of sour cherry fruit cultivars with the highest and the lowest content of phenolic acids.

Flavan 3-ols were not quantified in all cultivars (Figure 4), and were determined at a level from 8.7 mg/100 g FW (cv. “Mołodiożnaja”) to 63.1 mg/100 g FW (cv. “Wieluń 17”). Monomers and dimers were mainly detected (Supplementary Material Table S4), similar to the research of Karaaslan et al., Wojdyło et al. and others [6,20,36].

The total phenolic compounds by HPLC were from 96.56 mg/100 g FW in the “Nana” cultivar to 268.98 mg/100 g FW in the “Wieluń 17” cultivar. Other researchers determined 120.0–312.0 mg of phenolic compounds in 100 g of sour cherry fruit [27,35,37]. Due to the numerous cultivars and varieties, the composition and content of sour cherry constituents vary widely, and their level depends mainly on the cultivar, maturity, agronomic factors and climatic conditions [6,20,22].

### 2.4. Antioxidant Capacity

Antioxidant capacity was measured by three methods: DPPH, FRAP, and by determination of substances which react with Folin–Ciocalteu reagent (FCRS). FCRS—total phenolic content can be used as antioxidant capacity rather than total phenolic content, as other substances may interfere with the determination of the concentration of phenolic compounds (Table 4).

According to previous reports, the content of FCRS in sour cherries can be 74–754 mg GAE/100 g 100 g FW [2,7,22]. In our research, FCRS in sour cherry samples was from 205.7 mg GAE/100 g FW in the “Wieluń 17” variety to 495.2 mg GAE/100 g FW in the “Grosenkirch” cultivar. This is in agreement with the Phenol-Explorer 3.0 database [38]. Antioxidant capacity measured by the DPPH method was determined at the level of 510.6–984.8 µmol TE/100 g FW (“Paraszt Meggy” and “Grosenkirch” cultivars respectively). In the FRAP test, values from 1111.1 to 3065.8 µmol TE/100 g FW were obtained for the same cultivars—”Paraszt Meggy” and “Grosenkirch” respectively. Sour and sweet cherries and other fruits have a very different antioxidant capacity related mainly to the composition of phytochemicals.

It is well known that the antioxidant capacity is highly correlated with the phenolic content. Our study confirms this feature and we found a correlation of antioxidant capacity with phenolic content (0.64-0.83), especially anthocyanins and flavonols (Table 5). Similar antioxidant capacity values and relationships were found in the studies by Alba et al., Ferretti et al., Khoo et al. and others [1,7,10,18,22].

### 2.5. Principal Component Analysis

**Figure 5:**
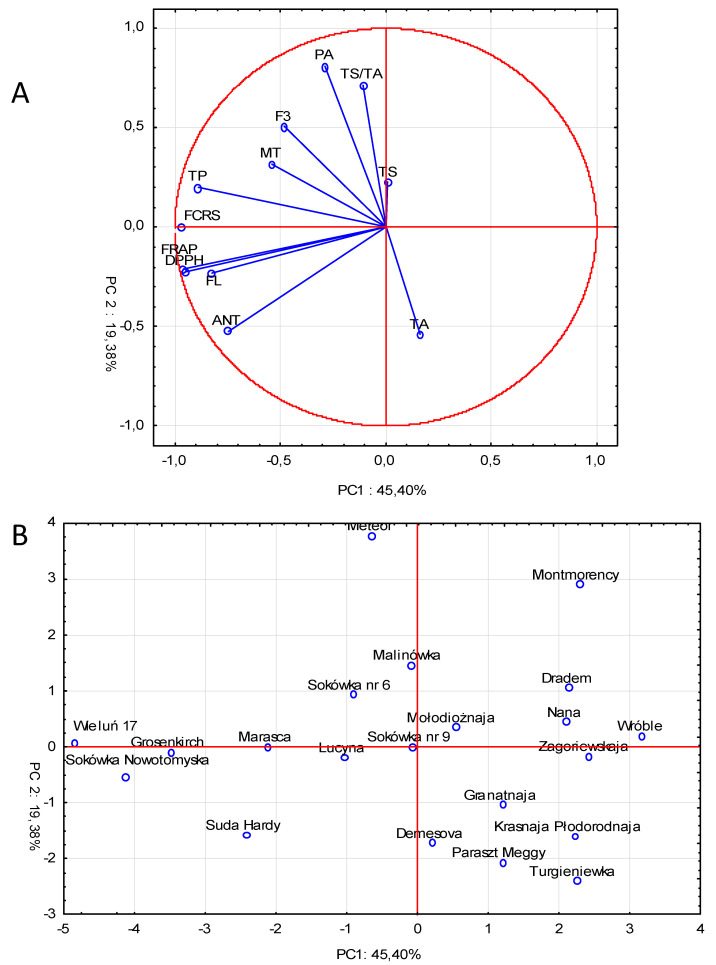
Principal component analysis (PCA) (**A**) score and loading plots (**B**) for sour cherry cultivars (ANT—anthocyanins, FL—flavonols, PA—phenolic acids, F3—flavan 3-ols, TP—total phenolic compounds, TA—total organic acids, TS—total sugars, TS/TA—total sugar to total acid ratio, MT—maturity harvest time, FCRS—Folin–Ciocalteu reacting substances.

The Principal Component Analysis (PCA) was applied to data to explain the relationships between sour cherry cultivars. The two first components explained 64.8% of the variation (Figure 5). The first component (45.4%) was associated with antioxidant activity (FCRS, DPPH, FRAP), anthocyanin (ANT) and flavonol (FL) content and maturity time (MT), while the second component (accounting for 19.4% of total variance) was mainly associated with phenolic acids (PA), flavan 3-ols (F3), organic acids (TA) and TS/TA ratio.

The high content of phenolic compounds was correlated with antioxidant capacity and with the time of maturity. Cultivars whose harvest maturity was medium to very late (“Wieluń 17”, “Grosenkirch”, “Suda Hardy”, “Marasca”, “Sokówka Nowotomyska”) contained more flavonols and anthocyanins and were characterized by higher antioxidant activity. In the second cluster were cultivars with low phenolic compound and antioxidant capacity and high content of organic acids (“Granatnaja”, “Krasnaja Płodorodnaja”, “Demesova”, “Paraszt Meggy” and “Turgieniewka”), whose harvest maturity was classified as early or early to medium. 

The results show that Polish genotypes such as “Wieluń 17”, “Sokówka Nowotomyska”, “Sokówka nr 9”, “Sokówka nr 6”, and “Lucyna” are characterized by a high content of bioactive compounds and also perform very well in terms of other characteristics, compared, for example, to the very popular and commonly studied Montmorency variety.

Our research indicates large biodiversity of the varieties grown in Poland, which is consistent with the objectives of sustainable agriculture.

## 3. Materials and Methods

### 3.1. Chemicals

Cyanidin 3 *O*-glucoside (Cy3-glc) was purchased from Extrasynthese (Lyon Nord, France). 5 *O*-caffeoylquinic acid (5-CQA, chlorogenic acid), quercetin 3 *O*-rutinoside, (+)-catechin, sugar standards, 1,1-diphenyl-2-picrylhydrazyl radical (DPPH); 2,4,6-tri(2-pyridyl)-s-triazine (TPTZ), 6-hydroxy-2,5,7,8-tetramethylchroman-2-carboxylic acid (Trolox); acetonitrile (gradient grade for HPLC), methanol, and formic acid (98–100%) were acquired from Sigma-Aldrich (Germany, Schnelldorf). Organic acids were purchased from Supelco Analytical (Bellefonte, PA, USA). Sulfuric acid, Folin–Ciocalteu reagent, hydrochloric acid, acetic acid, sodium carbonate and sodium acetate were purchased from Chempur (Piekary Śląskie, Poland).

All reagents were of analytical grade.

### 3.2. Plant Material

#### 3.2.1. Location

The sour cherry collection (150 m above sea level, 51°54′45.14″ N, 20°06′28.35″) were harvested in the Experimental Orchard in Dąbrowice, belonging to the Research Institute of Horticulture in Skierniewice.

#### 3.2.2. Plant Material Characteristics

Eighteen cultivars, “Demesova”, “Dradem”, “Granatnaja”, “Grosenkirch”, “Krasnaja Płodorodnaja”, “Lucyna”, “Malinówka”, “Marasca”, “Meteor”, “Mołodiożnaja”, “Montmorency”, “Nana”, “Paraszt Meggy”, “Sokówka Nowotomyska”, “Suda Hardy”, “Turgieniewka”, “Wróble”, “Zagoriewskaja”, and three local genotypes, “Sokówka nr 6”, “Sokówka nr 9”, “Wieluń 17”, were assessed in the study (Table 6).

Fruit samples were taken from well-fruiting trees, from the south-western side of each tree. The fruits were harvested at the “ready-to-eat” ripening stage, determined by its taste.

Genotypes were grafted on *Prunus mahaleb* seedlings. Trees grew on medium soil, without irrigation at a spacing 4.0 × 2.5 m. Ripened fruit was stored in a frozen state until analysis.

### 3.3. HPLC

#### 3.3.1. Preparation of Samples

Fully matured cherries were frozen at −20 °C and thawed directly before experiments. Thawed fruits of each cultivar (average portion approximately 200 g randomly chosen fruits) were depitted and homogenized using a blender (Zelmer, Poland).

#### 3.3.2. Samples for Phenolic Compounds and Antioxidant Capacity Determination

An amount of 5 g of the homogenate was extracted with 50 mL of 80% aqueous methanol (*v/v*) acidified with 1% HCl by ultrasonication for 20 min. The extract was centrifuged and diluted (re-distilled water with the ratio 1:1, *v/v*). For HPLC-PDA analysis the supernatant was filtered through a Hydrophilic PTFE 0.45 µm filter (Millex Samplicity Filter, Merck, Germany) and used for investigation of phenolic compounds and antioxidant capacity.

#### 3.3.3. Samples for the Determination of Sugars and Organic Acids

The determination of sugar and organic acid content was made by HPLC. Homogenized fruit (3 g) with about 20–30 mL of distilled water was sonicated for 15 min and next boiled for 30 min; then the sample was transferred to flasks (50 mL) with distilled water and centrifuged for 10 min. The supernatant (2.5 mL) was applied onto the Sep-Pak C-18 (containing 1 g of the carrier, Millipore Waters, Milford, MA, USA ) and eluted by water to Eppendorf tubes. The extract was filtered through 0.45 µm Millipore filters (Waters Millipore, Milford, MA, USA ).

#### 3.3.4. Phenolic Compounds

The analysis was previously described by Sokół-Łętowska et al. [39]. The HPLC-PDA analysis was performed using a Dionex (Germering, Germany) system equipped with the diode array detector model Ultimate 3000 System (Dionex, Germering, Germany), equiped with quaternary pump LPG-3400A), autosampler EWPS-3000SI and thermostatted column compartment TCC-3000SD, and controlled by Chromeleon v. 7.2 software (Thermo Scientific Dionex, Sunnyvale, CA, USA). The Cadenza Imtakt column CD-C18 (75 × 4.6 mm, 5 µm, Imtakt, Kyoto, Japan) was used. The mobile phase was composed of solvent A (4.5% aq. formic acid, *v/v*) and solvent B (100% acetonitrile). The elution system was as follows: 0–1 min 5% B in A, 20 min 25% B in A, 21 min 100% B, 26 min 100% B, 27 min 5% B in A. The flow rate of the mobile phase was 1.0 mL/min and the injection volume was 20 µL. The column was operated at 30 °C. Anthocyanins were detected at 520 nm, flavonols at 360 nm, phenolic acids and their derivatives at 320 nm, flavan 3-ols at 280 nm. All determinations were performed in triplicate. Results were expressed as mg/100 g FW of fruit. The content of anthocyanins, flavonols, flavanols, and hydroxycinnamic acids was calculated based on calibration curves determined experimentally (Table S5). The calibration curves were obtained on six levels of concentration of standard compounds (cyanidin 3-*O*-glucoside—Extrasynthese, Lyon Nord, France, quercetin 3-*O*-rutinoside, (+)-catechin and 5-*O*-caffeoylquinic acid—Sigma Aldrich, Steinheim, Germany), with three injections per level. Chromatogram peak areas were plotted against the known concentrations of the standard solutions. Linear regression equations were calculated by the least-squares method. As the correlation coefficients R^2^ were ≥0.999, the relations were considered linear and acceptable for quantifying the compounds. The content of anthocyanins was converted into cyanidin 3-*O*-glucoside, flavonols into quercetin 3-*O*-rutinoside, flavanols into (+)-catechin and hydroxycinnamic acids into 5-*O*-caffeoylquinic acid.

#### 3.3.5. Sugars

The extracts were filtered through a 0.45 µm Millipore filter (Waters, Milford, MA, USA). Samples were injected into a Unison UK Amino 3 µm column (3 mm × 250 mm) (Imtakt, Kyoto, Japan) liquid chromatograph. Determinations were carried out using an Merck-Hitachi L-7455 liquid chromatograph with an evaporative light scattering detector (ELSD; PL-ELS 1000, Polymer Laboratories, Darmstadt, Germany,) and the quaternary pump L-7100 equipped with the D-7000 HSM Multisolvent Delivery System and an L-7200 autosampler (Merck-Hitachi, Tokyo, Japan). Input parameters: evaporator temperature 80 °C; nebulizer temperature 80 °C; nitrogen flow—1.2 SLM. The elution was carried out under isocratic flow using an 85% acetonitrile solution at the flow rate of 0.7 mL/min and temperature 30 °C.

Sugars were identified by comparison with the standards of glucose, fructose, sucrose, arabinose, xylose and sorbitol. Concentrations of the standards used for the calibration curves were 0.5–5.0 mg/mL (Table S6). Linear regression equations were calculated by the least squares method. As the correlation coefficients R^2^ were ≥0.999, the relations were considered linear and acceptable for quantifying the compounds. All determinations were performed in triplicate. Results were expressed as g/100 g FW of fruit.

#### 3.3.6. Organic Acids

Organic acids were determined by the HPLC method, isocratically, using 0.001 N sulfuric acid, at 210 nm wavelength and flow of 0.6 mL/min [40]. The assay was performed using an HPLC instrument (Dionex Ultimate 3000 System, Dionex, Germering, Germany with the following devices: LPG-3400A pump, EWPS-3000SI autosampler TCC-3000SD column thermostat and the Chromeleon v. 7.2 computer software (Thermo Scientific Dionex, Sunnyvale, CA, USA). The separation was conducted on an Aminex HPH-87 H (300 × 7.8 mm) column with an IG Cation H (30 × 4.6 mm) precolumn from Bio-Rad (Hercules, CA USA), at a temperature of 65 °C. Organic acids were identified by comparison with the standards of malic acid, malonic acid, oxalic acid, shikimic acid and fumaric acid. Concentrations of the standards used for the calibration curves were mg/mL. Concentrations of the standards used for the calibration curves were 0.05–10.6 mg/mL (Table S7). Linear regression equations were calculated by the least-squares method. As the correlation coefficients R^2^ were ≥0.999, the relations were considered linear and acceptable for quantifying the compounds. All determinations were performed in triplicate. Results were expressed as mg/100 g FW of fruit.

### 3.4. Antioxidant Activity

#### 3.4.1. Content of Substances Which React with the Folic–Ciocalteu Reagent

Contents of substances which react with the Folic–Ciocalteu reagent (FCRS) were determined by the Folin–Ciocalteu method using gallic acid (GA) as a standard for the calibration curve. Sample (0.1 mL) was mixed in 4 mL couvettes with 0.2 mL of Folin–Ciocalteu reagent and 2 mL of H_2_O, and after 3 min, 1 mL 20% sodium carbonate. FCRS was determined after 1 h of incubation at room temperature in the dark. The results were read at 765 nm in a spectrophotometer (Shimadzu UV-2401 PC, Kyoto, Japan). All the determinations were performed in triplicate. The results of the assay were calculated and expressed as milligrams of GA equivalent (GAE) per 100 g FW of fruit.

#### 3.4.2. DPPH

The DPPH radical-scavenging activity of fruit was determined according to the method of Yen and Chen [41]. To each sample of appropriately diluted extract (0.5 mL), DPPH solution (approx. 0.04 mmol/L) and ethanol were added in the amount of 0.5 and 1.5 mL, respectively. The mixture was shaken and allowed to stand at room temperature for 10 min. Antioxidant capacity was measured by recording the absorbance at 517 nm using a spectrophotometer (Shimadzu UV-2401 PC). Ethanol was used as the blank. All the determinations were performed in triplicate. The antioxidant capacity of the fruit was expressed as Trolox equivalent antioxidant capacity. TE values were calculated and expressed as μmol Trolox equivalents (TE) per 100 g FW of fruit.

#### 3.4.3. FRAP

The antioxidant potential of samples was determined using a ferric reducing antioxidant power (FRAP) assay by Benzie and Strain [42] as a measure of antioxidant power. The FRAP reagent was prepared by mixing acetate buffer (300 mM, pH 3.6), a solution of 10 mM TPTZ in 40 mM HCl, and 20 mM FeCl_3_ at 10:1:1 (*v/v/v*). An aliquot (1.0 mL) of the diluted extract was added to 3 mL of FRAP solution, then the mixture was shaken and left at room temperature for 10 min. The absorbance was read at 593 nm after 10 min using a Shimadzu UV2401PC spectrophotometer. The standard curve was prepared using different concentrations of Trolox. The results of the assay were expressed in μmol Trolox equivalents (TE) per 100 g FW. All the determinations were performed in triplicates.

### 3.5. Statistical Analysis

Results were presented as the mean ± standard deviation of three replications. All statistical analyses were performed with Statistica version 13.1 (StatSoft, Tulsa, OK, USA). One-way analysis of variance (ANOVA) by Duncan’s test was used to compare the mean values. Differences were considered to be significant at α = 0.05.

Principal component analysis (PCA) was carried out to investigate the correlations between data on mean values of 21 sample cultivars and 12 variables: anthocyanins, flavonols, phenolic acids, flavan 3-ols, total phenolic compounds, total organic acids, total sugars, total sugars to total acids ratio, substances reacting with Folin–Ciocalteu reagent, DPPH test, FRAP test, harvest maturity.

## Figures and Tables

**Table 1:** Sugar content (g/100 g FW) in sour cherry fruit of 21 cultivars.

Cultivar	Glucose	Fructose	Sorbitol	Saccharose	Galactose	Xylose	Total
Demesova	4.85 ± 0.08 ^hg^	4.00 ± 0.06 ^ed^	2.74 ± 0.01 ^dc^	1.41 ± 0.03 ^ba^	nd	nd *	13.00 ± 0.33 ^d^
Dredem	5.37 ± 0.02 ^cb^	3.99 ± 0.08 ^ed^	2.40 ± 0.02 ^ih^	1.35 ± 0.01 ^edc^	nd	nd	13.11 ± 0.1 ^d^
Granatnaja	3.10 ± 0.01 ^m^	2.74 ± 0.01 ^k^	2.07 ± 0.01 ^k^	1.38 ± 0.01 ^dcb^	nd	1.36 ± 0.02 ^a^	10.65 ± 0.20 ^ih^
Grosenkirch	4.39 ± 0.07 ^kj^	3.60 ± 0.10 ^hg^	2.45 ± 0.03 ^hg^	nd	nd	nd	10.44 ± 0.15 ^i^
Krasnaja Płodorodnaja	4.43 ± 0.03 ^kj^	3.71 ± 0.03 ^gf^	2.42 ± 0.07 ^hg^	1.38 ± 0.03 ^cba^	nd	nd	11.93 ± 0.35 ^f^
Lucyna	5.68 ± 0.12 ^a^	4.36 ± 0.03 ^b^	2.47 ± 0.03 ^hgf^	1.38 ± 0.01 ^cba^	1.50 ± 0.00 ^a^	nd	15.38 ± 0.53 ^a^
Malinówka	4.91 ± 0.15 ^gfe^	4.00 ± 0.11 ^fe^	2.16 ± 0.07 ^k^	1.28 ± 0.04 ^hg^	nd	nd	12.34 ± 0.26 ^fe^
Marasca	4.74 ± 0.07 ^hgf^	3.89 ± 0.08 ^f^	2.34 ± 0.03 ^ji^	nd	nd	nd	10.97 ± 0.36 ^g^
Meteor	3.91 ± 0.01 ^l^	4.88 ± 0.15 ^a^	3.12 ± 0.08 ^a^	1.28 ± 0.02 ^hg^	1.39 ± 0.03 ^b^	nd	14.58 ± 0.05 ^b^
Mołodiożnaja	5.07±0.07 ^fed^	4.06 ± 0.13 ^d^	2.61 ± 0.04 ^fe^	1.40 ± 0.04 ^ba^	1.53 ± 0.01 ^a^	nd	14.67 ± 0.46 ^ba^
Montmorency	3.32 ± 0.09 ^m^	4.22 ± 0.13 ^dc^	2.75 ± 0.08 ^edc^	nd	Nd *	nd	10.29 ± 0.04 ^i^
Nana	4.20 ± 0.12 ^k^	3.55 ± 0.07 ^h^	1.93 ± 0.05 ^l^	1.03 ± 0.00 ^j^	nd	nd	10.71 ± 0.27 ^hg^
Paraszt Meggy	4.74 ± 0.08 ^ih^	4.09 ± 0.08 ^dc^	2.85 ± 0.03 ^c^	nd	nd	nd	11.68 ± 0.05 ^g^
Sokówka Nowotomyska	4.80 ± 0.16 ^hg^	3.31 ± 0.08 ^i^	2.29 ± 0.04 ^j^	1.32 ± 0.00 ^fed^	nd	1.31 ± 0.04 ^a^	13.03 ± 0.17 ^d^
Sokówka nr 6	5.18 ± 0.16 ^edcb^	4.09 ± 0.12 ^ed^	2.31 ± 0.01 ^ij^	1.31 ± 0.00 ^fe^	nd	nd	12.90 ± 0.02 ^de^
Sokówka nr 9	4.63 ± 0.11 ^ij^	3.87 ± 0.07 ^f^	2.52 ± 0.08 ^gf^	1.25 ± 0.01 ^h^	nd	nd	12.27 ± 0.27 ^fe^
Suda Hardy	5.29 ± 0.07 ^b^	4.35 ± 0.04 ^cb^	3.00 ± 0.03 ^b^	1.31 ± 0.04 ^fe^	nd	nd	13.95 ± 0.20 ^c^
Turgieniewka	3.67 ± 0.06 ^l^	3.01 ± 0.00 ^j^	1.93 ± 0.03 ^b^	nd	nd	nd	8.62 ± 0.12 ^j^
Wieluń 17	2.81 ± 0.00 ^n^	3.68 ± 0.09 ^gf^	2.36 ± 0.02 ^ij^	1.40 ± 0.02 ^a^	nd	nd	10.25 ± 0.22 ^i^
Wróble	5.02 ± 0.08 ^edc^	4.32 ± 0.05 ^cb^	2.71 ± 0.00 ^fed^	1.32 ± 0.02 ^gf^	nd	1.30 ± 0.04 ^b^	14.67 ± 0.38 ^b^
Zagoriewskaja	5.22 ± 0.01 ^dcb^	3.94 ± 0.11 ^f^	2.69 ± 0.09 ^edc^	1.24 ± 0.02 ^h^	nd	nd	13.09 ± 0.20 ^d^
Min-max	2.81–5.68	2.74–4.88	1.93–3.12	1.03–1.41	1.39–153	1.30–1.36	8.62–15.38
Average	4.54	3.89	2.48	1.32	1.47	1.33	12.31
Median	4.74	3.99	2.45	1.32	1.50	1.31	12.34

nd *—not detected. Values are expressed as mean ± standard deviation; the same superscripted letters within the same column means a statistically homogeneous group (*p* = 0.05).

**Table 2:** Organic acids content (mg/100 g FW) in sour cherry fruit of 21 cultivars.

Cultivar	Malic	Malonic	Oxalic	Shikimic	Fumaric	Total
Demesova	1858.77 ± 7.83 ^e^	270.64 ± 2.11 ^ji^	13.93 ± 0.32 ^b^	4.08 ± 0.01 ^a^	0.21 ± 0.00 ^e^	2147.63 ± 10.10 ^c^
Dredem	1810.91 ± 2.51 ^g^	313.44 ± 1.75 ^fe^	11.03 ± 0.26 ^e^	2.22 ± 0.02 ^h^	0.23 ± 0.00 ^d^	2137.82 ± 6.53 ^d^
Granatnaja	1602.67 ± 6.66 ^l^	299.88 ± 9.97 ^fe^	9.33 ± 0.39 ^g^	2.37 ± 0.01 ^f^	0.17 ± 0.01 ^ih^	1914.43 ± 7.30 ^j^
Grosenkirch	1684.85 ± 5.75 ^j^	295.01 ± 5.63 ^hg^	9.13 ± 0.16 ^h^	1.95 ± 0.07 ^j^	0.24 ± 0.01 ^d^	1991.17 ± 2.27 ^i^
Krasnaja Poodorodnaja	1976.15 ± 7.73 ^a^	258.40 ± 0.18 ^k^	6.74 ± 0.09 ^l^	1.21 ± 0.03 ^o^	0.15 ± 0.00 ^lkj^	2242.66 ± 4.11 ^b^
Lucyna	1636.94 ± 1.30 ^k^	372.38 ± 4.48 ^b^	8.54 ± 0.28 ^i^	1.89 ± 0.00 ^kj^	0.29 ± 0.01 ^b^	2020.03 ± 1.20 ^g^
Malinówka	1219.56 ± 1.03 ^p^	333.46 ± 2.36 ^c^	4.75 ± 0.12 ^o^	1.92 ± 0.05 ^jl^	0.35 ± 0.00 ^a^	1560.04 ± 5.87 ^n^
Marasca	1176.83 ± 1.63 ^r^	302.28 ± 0.43 ^gf^	5.22 ± 0.04 ^n^	1.74 ± 0.02 ^k^	0.15 ± 0.01 ^l^	1486.22 ± 2.65 ^o^
Meteor	1589.80 ± 4.88 ^ml^	406.53 ± 4.17 ^a^	9.08 ± 0.02 ^h^	2.72 ± 0.00 ^e^	0.16 ± 0.01 ^lkj^	2008.29 ± 6.66 ^h^
Mołodiożnaja	1798.26 ± 8.9 ^h^	318.22 ± 3.71 ^ed^	11.76 ± 0.01 ^d^	2.32 ± 0.02 ^gf^	0.19 ± 0.00 ^f^	2130.75 ± 3.69 ^d^
Montmorency	1027.35 ± 2.75 ^t^	260.13 ± 4.32 ^kj^	5.03 ± 0.02 ^n^	1.67 ± 0.04 ^l^	0.16 ± 0.01 ^kji^	1294.35 ± 2.63 ^p^
Nana	1599.93 ± 1.60 ^l^	279.38 ± 3.46 ^ih^	5.91 ± 0.17 ^m^	1.05 ± 0.06 ^p^	0.19 ± 0.00 ^g^	1886.46 ± 5.14 ^k^
Paraszt Meggy	1963.60 ± 3.79 ^b^	322.60 ± 0.23 ^dc^	10.16 ± 0.30 ^f^	3.86 ± 0.07 ^b^	0.26 ± 0.00 ^c^	2300.47 ± 1.37 ^a^
Sokówka Nowotomyska	1784.40 ± 3.80 ^h^	235.65 ± 5.42 ^l^	9.03 ± 0.12 ^h^	1.44 ± 0.03 ^n^	0.18 ± 0.00 ^hg^	2030.71 ± 0.10 ^g^
Sokówka nr 6	1579.90 ± 7.59 ^m^	317.01 ± 2.24 ^ed^	7.23 ± 0.10 ^k^	2.01 ± 0.11 ^i^	0.24 ± 0.01 ^d^	1906.39 ± 6.51 ^j^
Sokówka nr 9	1491.26 ± 3.70 ^o^	290.46 ± 1.23 ^gf^	23.44 ± 0.39 ^a^	2.26 ± 0.07 ^dg^	0.26 ± 0.00 ^c^	1807.67 ± 5.46 ^l^
Suda Hardy	1915.88 ± 4.74 ^d^	369.96 ± 3.14 ^b^	10.33 ± 0.27 ^f^	3.12 ± 0.02 ^c^	0.23 ± 0.00 ^d^	2299.52 ± 1.25 ^a^
Turgieniewka	1825.73 ± 6.33 ^f^	231.52 ± 6.88 ^l^	7.81 ± 0.10 ^j^	2.08 ± 0.00 ^ih^	0.20 ± 0.00 ^e^	2067.35 ± 9.93 ^f^
Wieluń 17	1528.21 ± 2.65 ^n^	260.08 ± 1.01 ^kj^	8.84 ± 0.23 ^i^	2.06 ± 0.05 ^ih^	0.20 ± 0.01^f^	1799.38 ± 5.08 ^m^
Wróble	1939.57 ± 3.65 ^c^	337.84 ± 5.81 ^c^	12.74 ± 0.20 ^c^	2.97 ± 0.02 ^d^	0.16 ± 0.01^lk^	2293.28 ± 1.02 ^a^
Zagoriewskaja	1748.39 ± 0.90 ^i^	330.58 ± 2.10 ^c^	6.84 ± 0.07 ^l^	1.55 ± 0.07 ^m^	0.16 ± 0.01 ^ji^	2087.52 ± 4.24 ^e^
Min-max	1027.35–1976.15	231.52–406.53	4.75–23.44	1.05–4.08	0.15–0.35	1294.35–2300.47
Average	1655.19	305.02	9.38	2.21	0.21	1972.01
Median	1684.85	302.28	9.03	2.06	0.20	2020.03

Values are expressed as mean ± standard deviation, the same superscripted letters within the same column means statistically homogenous group (*p* = 0.05).

**Table 3:** Phenolic compound content (mg/100 g FW) in sour cherry fruit of 21 cultivars.

Cultivar	Anthocyanins	Flavonols	Phenolic Acids	Flavan 3-ols	Total
Demesova	128.17 ± 1.06 ^b^	18.12 ± 0.24 ^e^	26.53 ± 0.09 ^n^		154.69 ± 0.97 ^f^
Dradem	17.97 ± 0.16 ^p^	15.84 ± 0.42 ^f^	44.00 ± 0.25 ^h^	40.10 ± 0.11 ^b^	117.91 ± 0.72 ^m^
Granatnaja	81.50 ± 1.46 ^h^	15.61 ± 0.11 ^gf^	35.10 ± 0.08 ^j^	2.87 ± 0.04 ^i^	119.45 ± 1.50 ^j^
Grosenkirch	121.38 ± 0.23 ^c^	19.65 ± 0.25 ^d^	76.25 ± 0.29 ^b^	35.44 ± 0.27 ^c^	252.72 ± 0.50 ^b^
Krasnaja Płodorodnaja	80.85 ± 0.55 ^h^	13.19 ± 0.23 ^i^	32.33 ± 0.04 ^k^		126.37 ± 0.74 ^l^
Lucyna	107.91 ± 0.56 ^e^	19.51 ± 0.21 ^d^	30.94 ± 0.06 ^l^	9.45 ± 0.23 ^g^	167.81 ± 0.52 ^g^
Malinówka	85.39 ± 1.47 ^g^	14.09 ± 0.54 ^ih^	57.13 ± 0.07 ^d^		1656.61 ± 1.94 ^h^
Marasca	112.95 ± 0.99 ^d^	21.60 ± 0.17 ^c^	44.45 ± 0.10 ^h^		178.99 ± 1.06 ^e^
Meteor	51.28 ± 0.71 ^l^	15.50 ± 0.10 ^hgf^	126.99 ± 0.94 ^a^	40.81 ± 1.15 ^b^	219.07 ± 2.80 ^d^
Mołodiożnaja	84.14 ± 1.52 ^g^	13.30 ± 0.18 ^i^	49.28 ± 0.81 ^f^	8.73 ± 0.15 ^g^	155.45 ± 2.37 ^h^
Montmorency	30.35 ± 0.59 ^o^	14.18 ± 2.62 ^ih^	73.35 ± 0.86 ^c^	26.94 ± 1.36 ^e^	144.82 ± 2.53 ^i^
Pamięci Wawilowa	33.38 ± 0.87 ^n^	11.09 ± 0.24 ^j^	46.54 ± 0.92 ^g^	5.55 ± 0.10 ^h^	96.56 ± 2.14 ^p^
Paraszt Meggy	100.30 ± 0.48 ^f^	16.16 ± 0.03 ^f^	28.27 ± 0.05 ^m^		144.73 ± 0.55 ^i^
Sokówka Nowotomyska	130.14 ± 0.13 ^ab^	23.18 ± 0.08 ^b^	53.71 ± 0.28 ^e^	33.82 ± 0.27 ^d^	240.85 ± 0.06 ^c^
Sokówka nr 6	67.72 ± 0.57 ^k^	14.39 ± 0.20 ^ihg^	44.84 ± 0.63 ^h^	26.69 ± 0.07 ^e^	153.64 ± 1.47 ^h^
Sokówka nr 9	81.02 ± 1.27 ^h^	14.06 ± 0.28 ^i^	35.91 ± 0.57 ^j^		130.99 ± 1.57 ^k^
Suda Hardy	107.06 ± 0.90 ^e^	25.08 ± 0.00 ^a^	37.42 ± 0.12 ^i^		169.55 ± 1.02 ^g^
Turgeniewka	74.61 ± 1.1 ^i^	14.32 ± 0.09 ^ihg^	16.56 ± 0.06 ^o^		105.50 ± 1.25 ^n^
Wieluń 17	131.28 ± 0.91 ^a^	20.73 ± 0.14 ^dc^	53.90 ± 0.19 ^e^	63.08 ± 0.54 ^a^	268.98 ± 0.32 ^a^
Wróble	48.49 ± 1.64 ^m^	10.81 ± 0.29 ^j^	29.13 ± 0.19 ^m^	13.31 ± 1.57 ^f^	101.74 ± 0.55 ^o^
Zagoriewskaja	72.28 ± 1.44 ^j^	10.41 ± 0.21 ^j^	44.23 ± 0.44 ^h^		126.92 ± 2.09 ^l^
Min-max	17.97–131.28	10.41–25.08	16.56–126.99	0.00–63.08	96.56–268.98
Average	83.24	16.23	46.99	14.61	161.07
Median	81.47	15.50	44.23	5.55	153.64

Values are expressed as mean ± standard deviation; the same superscripted letters within the same column means a statistically homogeneous group (*p* = 0.05).

**Table 4:** Antioxidant capacity (mg GAE/100 g FW, µmol TE/100 g FW) of sour cherry fruit of 21 cultivars.

Cultivar	FCRS mg GAE/100 g	DPPH µmol TE/100 g	FRAP µmol TE/100 g
Demesova	267.00 ± 0.81 ^ghij^	647.09 ± 0.64 ^e^	1461.00 ± 5.04 ^gf^
Dredem (Maćkowiaka)	262.75 ± 17.68 ^hij^	695.39 ± 1.72 ^j^	1673.66 ± 22.94 ^k^
Granatnaja	250.46 ± 11.47 ^hg^	675.22 ± 13.51 ^f^	1681.80 ± 17.59 ^h^
Grosenkirch	495.20 ± 5.31 ^b^	984.81 ± 11.95 ^b^	3065.78 ± 36.24 ^b^
Krasnaja Płodorodnaja	239.35 ± 18.21 ^hij^	552.32 ± 9.09 ^h^	1183.87 ± 7.80 ^i^
Lucyna	244.17 ± 2.89 ^e^	739.90 ± 4.92 ^c^	1853.96 ± 2.41 ^d^
Malinówka	238.44 ± 1.31 ^f^	634.20 ± 6.63 ^e^	1539.87 ± 54.43 ^f^
Marasca	432.29 ± 10.95 ^cd^	910.45 ± 11.08 ^b^	2650.47 ± 42.80 ^c^
Meteor	245.36 ± 3.51 ^f^	631.65 ± 1.36h ^g^	1472.12 ± 29.10 ^i^
Mołodiożnaja	412.35 ± 6.92 ^f^	907.86 ± 11.02 ^e^	2613.64 ± 0.31 ^g^
Montmorency	309.18 ± 3.08 ^ijk^	655.14 ± 5.77 ^k^	1542.40 ± 28.06 ^kl^
Nana	340.16 ± 16.41 ^g^	826.61 ± 17.55 ^h^	2174.62 ± 4.42 ^i^^j^
Paraszt Meggy	227.71 ± 0.94 ^ghi^	510.62 ± 0.69 ^gf^	1111.10 ± 88.16 ^h^
Sokówka Nowotomyska	220.22 ± 3.81 ^a^	607.84 ± 3.12 ^a^	1444.57 ± 55.15 ^a^
Sokówka nr 6	376.70 ± 14.46 ^d^	804.12 ± 6.00 ^cd^	2197.94 ± 52.67 ^d^
Sokówka nr 9	475.68 ± 0.09 ^e^	975.62 ± 8.56 ^d^	3028.11 ± 24.87 ^e^
Suda Hardy	306.71 ± 16.67 ^bc^	749.08 ± 17.19 ^b^	1930.53 ± 59.23 ^b^
Turgieniewka	342.96 ± 5.87 ^ghi^	802.01 ± 7.78 ^h^	2082.88 ± 33.92 ^ij^
Wieluń 17	205.71 ± 0.66 ^a^	520.98 ± 29.49 ^a^	1045.68 ± 31.84 ^a^
Wróble	392.83 ± 19.05 ^k^	891.29 ± 6.52 ^k^	2501.18 ± 1.27 ^l^
Zagoriewskaja	298.34 ± 12.74 ^k^	726.10 ± 3.95 ^i^	1792.50 ± 23.78 ^j^
Min-max	205.71–495.20	510.62–984.81	1045.68–3065.78
Average	313.50	735.63	1907.03
Median	298.34	726.10	1792.50

Values are expressed as mean ± standard deviation; the same superscripted letters within the same column means a statistically homogeneous group (*p* = 0.05).

**Table 5:** Correlation coefficients between phenolic compounds and antioxidant capacity tests.

	ANT	FL	PA	F3	TP	FCRS	DPPH	FRAP
ANT	1.00							
FL	0.70	1.00						
PA	−0.18	0.05	1.00					
FS	−0.02	0.26	0.58	1.00				
SP	0.64	0.68	0.57	0.65	1.00			
FCRS	0.66	0.77	0.24	0.48	0.80	1.00		
DPPH	0.83	0.80	0.04	0.23	0.73	0.94	1.00	
FRAP	0.83	0.81	0.06	0.30	0.76	0.96	0.99	1.00

ANT—anthocyanins, FL—flavonols, PA—phenolic acids, F3—flavan 3-ols, TP—total phenolics, *p* = 0.05.

**Table 6:** Plant material characteristics.

Cultivar	Origin		Fruits				
	Parents	Country **	Harvest Maturity		Weight [g]	Skin Colour	Juice Colour
“Demesova”	unknown	probably the Czech Republic	medium	12–16.07	4.5–5.0	black	black red
“Dradem”	“Łutówka” x “Northstar”	Poland	medium to late	15–20.07	5.0–5.5	light red	colourless
“Granatnaja”	unknown	former USSR	medium	05–15.07	5.3–5.6	red	red
“Grosenkirch”	unknown	probably. the Netherlands	late	16–22.07	5.5–5.9	red	red
“Krasnaja Płodorodnaja”	unknown	former USSR	early to medium	02–09.07	4.6–4.9	dark red	dark red
“Lucyna”	“Łutówka” x “Schirpotreb”	Poland	medium to late	10–20.07	5.1–5.6	dark red	red
“Malinówka”	unknown	former USSR	late	15–25.07	4.8–5.3	light red	red
“Marasca”	unknown	former Yugoslavia	medium to late	10–20.07	2.7–3.2	dark red	dark red
“Meteor”	“Montmorency” x (“Vladimir” x “Shubianka”)	USA	late	15–25.07	5.2–5.6	light red	pink
“Mołodiożnaja”	unknown	Ukraine	medium to late	05–20.07	4.6–5.0	dark red	dark red
“Montmorency”	unknown	USA	early to medium	01–15.07	4.5–5.0	light red	colourless
“Nana”	“Crisana” x “Morella Neagra”	Romania	late	15–25.07	4.9–5.3	red	red
“Paraszt Meggy”	unknown	probably Hungary	medium	03–15.07	4.6–5.0	dark red	dark red
“Sokówka Nowotomyska”	unknown	Poland	medium to late	10–25.07	3.8–4.2	dark red	dark red
“Sokówka nr 6” *	unknown	Poland	late to very late	20–28.07	4.5–4.9	dark red	red
“Sokówka nr 9” *	unknown	Poland	late	15–25.07	4.0–4.4	red	red
“Suda Hardy”	probably seedling cv “Łutówka”	USA	late	15–25.07	4.6–4.9	red	red
“Turgieniewka”	seedling cv “Żukowskaja”	Russia	medium	05–15.07	5.2–5.6	red	red
“Wieluń 17” *	unknown	Poland	late	15–25.07	2.9–3.7	dark red	dark red
“Wróble”	unknown	prob. the Chech Republic	medium to late	10–20.07	5.0–5.5	light red	light red
“Zagoriewskaja”	unknown	prob. Russia	medium	05–15.07	4.8–5.6	dark red	light red

* local genotype, ** country of origin.

## Supplementary Materials

The following are available online, Table S1: Anthocyanin profile (mg/100 g FW) in fruit of 21 sour cherry cultivars; Table S2: Flavonol profile (mg/100 g FW) in fruit of 21 sour cherry cultivars; Table S3: Phenolic acid profile (mg/100 g FW) in fruit of 21 sour cherry cultivars; Table S4: Flavan 3-ol profile (mg/100 g FW) in fruit of 21 sour cherry cultivars

## References

[1] Alba C.M.A., Daya M., Franck C. (2019). Tart cherries and health: Current knowledge and need for a better understanding of the fate of phytochemicals in the human gastrointestinal tract. Crit. Rev. Food Sci. Nutr..

[2] Blando F., Oomah B.D. (2019). Sweet and sour cherries: Origin, distribution, nutritional composition and health benefits. Trends Food Sci. Technol..

[3] Kelley S.D., Adkins Y., Laugero D.K. (2018). A Review of the health benefits of cherries. Nutrients.

[4] Viljevac-Vuletić M., Dugalić K., Mihaljević I., Tomaš V., Vuković D., Zdunić Z., Puškar B., Jurković Z. (2017). Season, location and cultivar influence on bioactive compounds of sour cherry fruits. Plant Soil Env..

[5] Neveu V., Perez-Jiménez J., Vos F., Crespy V., du Chaffaut L., Mennen L., Knox C., Eisner R., Cruz J., Wishart D. (2010). Phenol-Explorer: An online comprehensive database on polyphenol contents in foods. Database.

[6] Wojdyło A., Nowicka P., Laskowski P., Oszmiański J. (2014). Evaluation of sour cherry (*Prunus cerasus* L.) fruits for their polyphenol content, antioxidant properties, and nutritional components. J. Agric. Food Chem..

[7] Khoo G.M., Clausen M.R., Pedersen B.H., Larsen E. (2011). Bioactivity and total phenolic content of 34 sour cherry cultivars. J. Food Compos. Anal..

[8] Najafzadeh R., Arzani K., Bouzari N., Hashemi J. (2014). Identification of new Iranian sour cherry genotypes with enhanced fruit quality parameters and high antioxidant properties. N. Z. J. Crop Hortic. Sci..

[9] Feldmane D. Investigation of the biochemical composition of sour cherry (*Prunus cerasus* L.) fruits grown in Latvia. Proceedings of the International Scientific Conference Sustainable Fruit Growing: From Plant To Product.

[10] Alrgei H.O.S., Dabić D.Č., Natić M.M., Rakonjac V.S., Milojković-Opsenica D., Tešić Ž.L., Fotirić Akšić M.M. (2016). Chemical profile of major taste- and health-related compounds of Oblačinska sour cherry. J. Sci. Food Agric..

[11] FAOSTAT. http://www.fao.org/faostat/en/#data/QC.

[12] Papp N., Szilvássy B., Abrankó L., Szabó T., Pfeiffer P., Szabó Z., Nyéki J., Ercisli S., Stefanovits-Bányai É., Hegedűs A. (2010). Main quality attributes and antioxidants in Hungarian sour cherries: Identification of genotypes with enhanced functional properties. Int. J. Food Sci. Technol..

[13] Grafe C., Schuster M. (2014). Physicochemical characterization of fruit quality traits in a German sour cherry collection. Sci. Hortic..

[14] Kucharska A.Z., Sokół-Łętowska A., Piórecki N. (2011). Morphological physical & chemical, and antioxidant profiles of polish varieties of cornelian cherry fruit (*Cornus mas* L.). Zywnosc-Nauka Technol. Jakosc.

[15] Kucharska A.Z. (2020). Związki Aktywne Owoców Derenia (Cornus mas L.).

[16] Nawirska-Olszańska A., Kolniak-Ostek J., Oziembłowski M., Ticha A., Hyšpler R., Zadak Z., Židová P., Paprstein F. (2017). Comparison of old cherry cultivars grown in Czech Republic by chemical composition and bioactive compounds. Food Chem..

[17] Usenik V., Fabčič J., Štampar F. (2008). Sugars, organic acids, phenolic composition and antioxidant activity of sweet cherry (*Prunus avium* L.). Food Chem..

[18] Cao J., Jiang Q., Lin J., Li X., Sun C., Chen K. (2015). Physicochemical characterisation of four cherry species (Prunus spp.) grown in China. Food Chem..

[19] McCune L.M., Kubota C., Stendell-Hollis N.R., Thomson C.A. (2010). Cherries and Health: A Review. Crit. Rev. Food Sci. Nutr..

[20] Borowy A., Chrzanowska E., Kapłan M. (2018). Comparison of three sour cherry cultivars grown in central-eastern Poland. Acta Sci. Pol. Hortorum Cultus.

[21] Antoniewska A., Rutkowska J., Adamska A. (2017). Charakterystyka owoców pigwowca japońskiego oraz ich zastosowanie w przemyśle spożywczym. ŻYWNOŚĆ Nauka Technol. Jakość.

[22] Ferretti G., Bacchetti T., Belleggia A., Neri D. (2010). Cherry antioxidants: From farm to table. Molecules.

[23] Milosevic T., Milosevic N., Mladenovic J. (2019). Tree vigor, yield, fruit quality, and antioxidant capacity of apple (*Malus* × *domestica* Borkh.) influenced by different fertilization regimes: Preliminary results. Turk. J. Agric. For..

[24] Mratinić E., Popovski B., Milošević T., Popovska M. (2012). Impact of harvest time on the main agronomic and fruit quality traits of three apricot cultivars. Int. J. Fruit Sci..

[25] Yan L., Zhou X., Shi L., Shalimu D., Ma C., Liu Y. (2017). Phenolic profiles and antioxidant activities of six Chinese pomegranate (*Punica granatum* L.) cultivars. Int. J. Food Prop..

[26] Picariello G., De Vito V., Ferranti P., Paolucci M., Volpe M.G. (2016). Species- and cultivar-dependent traits of *Prunus avium* and *Prunus cerasus* polyphenols. J. Food Compos. Anal..

[27] Jakobek L., Šeruga M., Šeruga B., Novak I., Medvidović-Kosanović M. (2009). Phenolic compound composition and antioxidant activity of fruits of Rubus and Prunus species from Croatia. Int. J. Food Sci. Technol..

[28] Nowicka P., Wojdylo A., Lech K., Figiel A. (2015). Chemical composition, antioxidant capacity, and sensory quality of dried sour cherry fruits pre-dehydrated in fruit concentrates. Food Bioprocess Technol..

[29] Mulabagal V., Lang G.A., DeWitt D.L., Dalavoy S.S., Nair M.G. (2009). Anthocyanin content, lipid peroxidation and cyclooxygenase enzyme inhibitory activities of sweet and sour Cherries. J. Agric. Food Chem..

[30] Šimunić V., Kovač S., Gašo-Sokač D., Pfannhauser W., Murkovic M. (2005). Determination of anthocyanins in four Croatian cultivars of sour cherries (*Prunus cerasus*). Eur. Food Res. Technol..

[31] Kucharska A.Z., Szumny A., Sokół-Łętowska A., Piórecki N., Klymenko S.V. (2015). Iridoids and anthocyanins in cornelian cherry (*Cornus mas* L.) cultivars. J. Food Compos. Anal..

[32] Blando F., Gerardi C., Nicoletti I. (2004). Sour Cherry (*Prunus cerasus* L) anthocyanins as ingredients for functional foods. J. Biomed. Biotechnol..

[33] Homoki J.R., Nemes A., Fazekas E., Gyémánt G., Balogh P., Gál F., Al-Asri J., Mortier J., Wolber G., Babinszky L. (2016). Anthocyanin composition, antioxidant efficiency, and α-amylase inhibitor activity of different Hungarian sour cherry varieties (*Prunus cerasus* L.). Food Chem..

[34] Filimon R.V., Beceanu D., Niculaua M., Arion C. (2011). Study on the anthocyanin content of some sour cherry varieties grown in Iaşi area, Romania. Cercet. Agron. Mold..

[35] Levaj B., Dragović-Uzelac V., Ganić K.K., Banović M., Kovačević D.B. (2010). Polyphenols and volatiles in fruits of two sour cherry cultivars, some berry fruits and their jams. Food Technol. Biotechnol..

[36] Karaaslan M., Yılmaz F.M., Karaaslan A., Vardin H. (2016). Synthesis and accumulation of anthocyanins in sour cherries during ripening in accordance with antioxidant capacity development and chalcone synthase expression. Eur. Food Res. Technol..

[37] Kim D.-O., Heo H.J., Kim Y.J., Yang H.S., Lee C.Y. (2005). Sweet and sour cherry phenolics and their protective effects on neuronal cells. J. Agric. Food Chem..

[38] Rothwell J.A., Perez-Jimenez J., Neveu V., Medina-Remón A., M’Hiri N., García-Lobato P., Manach C., Knox C., Eisner R., Wishart D.S. (2013). Phenol-Explorer 3.0: A major update of the Phenol-Explorer database to incorporate data on the effects of food processing on polyphenol content. Database.

[39] Sokół-Łętowska A., Kucharska A.Z., Szumny A., Wińska K., Nawirska-Olszańska A. (2018). Phenolic composition stability and antioxidant activity of sour cherry liqueurs. Molecules.

[40] Sturm K., Koron D., Stampar F. (2003). The composition of fruit of different strawberry varieties depending on maturity stage. Food Chem..

[41] Yen G.-C., Chen H.-Y. (1995). Antioxidant activity of various tea extracts in relation to their antimutagenicity. J. Agric. Food Chem..

[42] Benzie I.F.F., Strain J.J. (1996). The Ferric Reducing Ability of Plasma (FRAP) as a Measure of “Antioxidant Power”: The FRAP Assay. Anal. Biochem..

