# Supplementary material for: Chemical Composition of 21 Cultivars of Sour Cherry (Prunus cerasus) Fruit Cultivated in Poland
Source: Molecules. 2020 Oct 8;25(19):4587. doi: 10.3390/molecules25194587 (PMC7582279; doi:10.3390/molecules25194587)
Supplement: Supplementary file 1 [file molecules-25-04587-s001.pdf]

Table S1. Anthocyanin profile (mg/100g FW) in fruit of 21 sour cherry cultivars.

Cultivar	Cyanidin 3-sophoroside	Cyanidin 3-glucosyl rutinoside	Cyanidin 3-glucoside	Cyanidin 3-galactoside	Cyanidin 3-rutinoside	Cyanidin derivative	Total mg/100g
Demesova	0.21	0.00	7.03	0.00	120.39	0.54	128.17
Dradem	0.21	12.33	0.14	0.17	4.68	0.45	17.97
Granatnaja	0.13	0.00	2.68	0.00	76.42	2.25	81.47
Grosenkirche	10.44	95.93	1.48	1.09	11.79	0.65	121.38
Krasnaja Płodorodnaja	6.45	59.65	1.31	0.47	12.42	0.55	80.85
Lucyna	7.31	71.77	2.44	1.93	23.36	1.10	107.91
Malinówka	4.63	55.04	2.25	0.42	21.68	1.38	85.39
Marasca	9.21	73.99	3.23	2.16	22.76	1.61	112.95
Meteor	1.44	38.24	0.38	0.33	10.00	0.88	51.28
Mołodiożnaja	4.61	62.64	0.81	1.62	12.73	1.74	84.14
Montmorency	0.19	21.38	0.38	0.23	7.39	0.78	30.35
Nana	1.63	23.11	0.26	0.51	6.50	1.38	33.38
Paraszt Meggy	6.96	65.84	1.60	1.92	22.25	1.74	100.30
Sokówka Nowotomyska	9.90	86.61	2.74	2.62	26.47	1.80	130.14
Sokówka nr 6	5.09	45.80	0.81	1.33	13.48	1.21	67.72
Sokówka nr 9	8.17	54.89	1.10	1.55	14.27	1.05	81.02
Suda Hardy	6.48	70.88	2.07	2.01	24.06	1.55	107.06
Turgieniewka	4.21	50.04	1.35	1.18	15.86	1.99	74.61
Wieluń 17	10.22	86.20	3.41	2.63	26.80	2.02	131.28
Wróble	0.59	34.62	0.38	0.48	11.14	1.28	48.49
Zagoriewskaja	2.99	53.32	0.94	0.65	11.76	2.63	72.28
% in Total anthocyanins	0.2-10.1	0.0-79.0	0.7-3.3	0.0-2.0	9.7-93.8	0.4-4.1	
Average %	5.2	62.8	1.8	1.3	27.0	1.9	

Table S2. Flavonols content (mg/100g FW) in fruit of 21 sour cherry cultivars.

Cultivar	Kaempherol derivative 1	Kaempherol derivative 2	Quercetin derivative	Quercetin 3-rutinoside	Isorhamnetin 3-rutinoside	Total mg/100g
Demesova	nd	11.08	0.00	7.04	0,00	18.12
Dradem	1.09	0.33	3.01	6.38	5.03	15.84
Granatnaja	nd	5.98	0.00	7.63	2.01	15.61
Grosenkirche	7.33	1.36	5.54	2.72	2.70	19.65
Krasnaja Podorodnaja	4.96	1.36	3.19	2.15	1.53	13.19
Lucyna	6.46	2.22	4.44	3.73	2.65	19.51
Malinówka	4.31	1.78	2.65	3.15	2.20	14.09
Marasca	6.44	2.53	4.27	4.34	4.02	21.60
Meteor	2.79	nd	1.34	6.00	5.37	15.50
Mołodiożnaja	4.55	0.81	3.21	1.51	3.22	13.30
Montmorency	1.59	1.71	2.54	4.17	4.17	14.18
Pamięci Wawiłowa	1.85	0.54	0.96	2.76	4.98	11.09
Paraszt Meggy	5.83	2.20	3.71	2.38	2.03	16.16
Sokówka Nowotomyska	7.94	2.88	5.39	3.81	3.16	23.18
Sokówka nr 6	4.33	1.45	2.91	2.79	2.92	14.39
Sokówka nr 9	5.42	1.90	3.52	1.70	1.52	14.06
Suda Hardy	7.03	2.66	3.96	5.44	6.00	25.08
Turgeniewka	4.53	1.36	3.68	2.31	2.44	14.32
Wieluń 17	5.79	2.32	4.99	4.05	3.57	20.73
Wróble	2.61	0.61	0.82	3.41	3.36	10.81
Zagoriewskaja	4.78	0.97	2.30	0.84	1.51	10.41
% in Total flavonols	0.0-85.0	0.0-38.3	0.0-45.0	15.0-48.9	0.0-54.0	
Average %	39.3	16.9	26.5	30.0	26.7	

*nd-not detected

Table S3 Phenolic acids profile (mg/100g FW) in fruit of 21 sour cherry cultivars.

Cultivar	Neochlorogenic acid	p-coumaric acid	Chlorogenic acid	Dicaffeoylquinic acid	Total mg100/g
Demesova	17.40	1.67	6.50	0.96	26.53
Dradem	16.35	15.98	10.52	1.15	44.00
Granatnaja	12.83	13.56	7.58	1.13	35.10
Grosenkirche	15.11	50.69	9.83	0.62	76.25
Krasnaja Podorodnaja	7.63	15.27	7.94	1.49	32.33
Lucyna	6.48	15.04	8.17	1.25	30.94
Malinówka	10.37	22.77	21.32	2.67	57.13
Marasca	9.93	20.31	13.12	1.09	44.45
Meteor	16.63	45.70	60.33	4.33	126.99
Mołodiożnaja	14.97	20.34	12.40	1.57	49.28
Montmorency	19.07	31.00	20.28	3.00	73.35
Pamięci Wawiłowa	34.47	2.95	7.83	1.29	46.54
Paraszt Meggy	5.24	12.71	9.40	0.91	28.27
Sokówka Nowotomyska	10.98	25.97	15.49	1.27	53.71
Sokówka nr 6	11.83	14.75	16.35	1.91	44.84
Sokówka nr 9	9.12	11.72	13.64	1.43	35.91
Suda Hardy	8.07	15.05	13.13	1.16	37.42
Turgeniewka	7.58	6.44	1.88	0.65	16.56
Wieluń 17	11.74	25.74	15.49	0.94	53.90
Wróble	6.16	11.50	9.69	1.79	29.13
Zagoriewskaja	15.89	24.40	2.99	0.95	44.23
% in Total phenolic acids	13.0-74.1	6.3-66.5	6.8-47.5	0.8-6.1	
Average %	29.7	39.8	27.1	3.4	

Table S4 Flavan 3-ols content in sour cherry cultivars.

Cultivar	Procyanidin B1	(-)Epicatechin	Procyanidin C1	Total mg100/g
Demesova	0.00	0.00	0.00	0.00
Dradem	22.36	13.86	3.88	40.10
Granatnaja	2.87	0.00	0.00	2.87
Grosenkirche	0.00	28.22	7.22	35.44
Krasnaja Podorodnaja	0.00	0.00	0.00	0.00
Lucyna	9.45	0.00	0.00	9.45
Malinówka	0.00	0.00	0.00	0.00
Marasca	0.00	0.00	0.00	0.00
Meteor	31.55	0.00	9.26	40.81
Mołodiożnaja	0.00	8.73	0.00	8.73
Montmorency	21.04	0.00	5.91	26.94
Pamięci Wawiłowa	0.00	5.55	0.00	5.55
Paraszt Meggy	0.00	0.00	0.00	0.00
Sokówka Nowotomyska	0.00	25.22	8.60	33.82
Sokówka nr 6	0.00	19.58	7.11	26.69
Sokówka nr 9	0.00	0.00	0.00	0.00
Suda Hardy	0.00	0.00	0.00	0.00
Turgeniewka	0.00	0.00	0.00	0.00
Wieluń 17	27.69	26.69	8.70	63.08
Wróble	0.00	11.91	1.40	13.31
Zagoriewskaja	0.00	0.00	0.00	0.00
% in Total flavan 3-ols	0.0-100.0	0.0-100.0	0.0-26.6	
Average %	21.7	28.3	7.2	

Table S5. Linear range, calibration curve, correlation coefficient R data for phenolic compounds standards.

Standard	Linear range (µg/mL)	$\lambda^{1)}_{det}$ (nm)	Calibration curve	Correlation coefficient R^2
Cyanidin 3-*O*-glucoside	10-75	520	$y^{2)}=1.1784x^{3)} - 0.9237$	0.9992
Quercetin 3-*O*-rutinoside	20-200	360	y=0.468x + 1.2227	0.9992
5-*O*-Caffeoylquinic acid	20-300	320	y=1.0813x + 1.2424	0.9998
(+)-Catechin	20-200	280	y=0.2388x + 0.0617	0.9995

1) λ_{det} – detection wavelength in quantification process

2) y – peak area

3) x- concentration

Table S6. Linear range, calibration curve, correlation coefficient R data for sugars standards.

Standard	Linear range (µg/mL)	Calibration curve	Correlation coefficient R^2
xylose	0.5–5.0	y[1]=1612.374 x[2] + 677132.9	0.9998
fructose	0.5–5.0	y=2065.167 x + 1152560	0.9998
sorbitol	0.5–5.0	y=1977.128 x + 1027388	0.9999
glucose	0.5–5.0	y=1547.412 x + 822839.6	0.9998
galactose	0.5–5.0	y=1118.514 x + 513812.6	0.9999
saccharose	0.5–5.0	y=1634.031 x + 693632.9	0.9998

1) y – peak area

2) x- concentration

Table S7. Linear range, calibration curve, correlation coefficient R data for organic acids standards.

Standard	Linear range (µg/mL)	$\lambda^{1)}{}_{det}$ (nm)	Calibration curve	Correlation coefficient R^2
Oxalic acid	96-960	210	y = 0.4982x + 6.2563	0.9997
Shikimic acid	49-490	210	y=1.5406x + 18.051	0.9991
Malic acid	1048-5240	210	y = 0.0289x + 0.4407	0.9999
Fumaric acid	49-246	210	y = 3.6287x + 33.26	0.9990
Malonic acid	530-10588	210	y = 0.032x + 0.896	0.9999

1) λ_{det} – detection wavelength in quantification process

2) y – peak area

3) x- concentration
